# Healthy lifestyle and risk of breast cancer for indigenous and non-indigenous women in New Zealand: a case control study

**DOI:** 10.1186/1471-2407-14-12

**Published:** 2014-01-10

**Authors:** Fiona McKenzie, Lis Ellison-Loschmann, Mona Jeffreys, Ridvan Firestone, Neil Pearce, Isabelle Romieu

**Affiliations:** 1 International Agency for Research on Cancer, Lyon, France; 2 Centre for Public Health Research, Massey University, Wellington, New Zealand; 3 School of Social and Community Medicine, University of Bristol, Bristol, UK; 4 London School of Hygiene and Tropical Medicine, London, UK

**Keywords:** Breast cancer, Health index, Lifestyle, Ethnicity, Indigenous health

## Abstract

**Background:**

The reasons for the increasing breast cancer incidence in indigenous Māori compared to non-Māori New Zealand women are unknown. The aim of this study was to assess the association of an index of combined healthy lifestyle behaviours with the risk of breast cancer in Māori and non-Māori women.

**Methods:**

A population-based case–control study was conducted, including breast cancer cases registered in New Zealand from 2005–2007. Controls were matched by ethnicity and 5-year age bands. A healthy lifestyle index score (HLIS) was generated for 1093 cases and 2118 controls, based on public health and cancer prevention recommendations. The HLIS was constructed from eleven factors (limiting red meat, cream, and cheese; consuming more white meat, fish, fruit and vegetables; lower alcohol consumption; not smoking; higher exercise levels; lower body mass index; and longer cumulative duration of breastfeeding). Equal weight was given to each factor. Logistic regression was used to estimate the associations between breast cancer and the HLIS for each ethnic group stratified by menopausal status.

**Results:**

Among Māori, the mean HLIS was 5.00 (range 1–9); among non-Māori the mean was 5.43 (range 1.5-10.5). There was little evidence of an association between the HLIS and breast cancer for non-Māori women. Among postmenopausal Māori, those in the top HLIS tertile had a significantly lower odds of breast cancer (Odds Ratio 0.47, 95% confidence interval 0.23-0.94) compared to those in the bottom tertile.

**Conclusion:**

These findings suggest that healthy lifestyle recommendations could be important for reducing breast cancer risk in postmenopausal Māori women.

## Background

The burden of breast cancer is considerable in New Zealand; women have an age standardised incidence rate of 89.4 per 100,000 compared with 84.8 in Australia, and 76.0 in the USA [1]. Furthermore, rates are highest among Māori women [2]. Māori are the indigenous population of New Zealand, comprising approximately 15% of the total population. People with ancestry originating from the United Kingdom and Europe make up about 77% of the population, while the remaining major ethnic groupings comprise those from Asian countries (approximately 10%) and from the Pacific Islands (approximately 7%) [3]. These figures add to more than 100%, as New Zealanders can identify with more than one ethnicity.

The incidence of breast cancer in Māori women appears to be increasing faster than in other ethnic groups. The age standardised breast cancer rate for European/Other women rose from 114 to 170 per 100,000 women per year from 1981–86 to 2001–04. Over the same period the corresponding rates for Māori rose from 123 to 210 per 100,000 women [4]. Since 1998, New Zealand has had a free national breast screening programme, which currently screens women aged between 45 and 69 every two years. Māori women have lower breast screening uptake than non-Māori women in New Zealand [5], and the reasons for the unequal and increasing breast cancer incidence among Māori are unknown, and virtually unexplored.

There is considerable evidence regarding individual lifestyle factors and breast cancer risk [6-13]. There is also a growing body of evidence relating various combined lifestyle factors, or patterns of behaviour, to cardiovascular disease [14,15] and diabetes [16], and more recently, to cancer [17,18]. The magnitude of the benefits of adhering to a healthy lifestyle has recently been highlighted in relation to breast cancer risk for Mexican women. Sanchez-Zamorano and colleagues observed a 50% lower risk for premenopausal and 80% for postmenopausal women, when comparing breast cancer risk in the highest quintile of a healthy lifestyle index to the lowest quintile [19].

The aim of this study was to assess the combined effect of healthy lifestyle behaviours on the risk of breast cancer, separately for Māori and non-Māori women. A “healthy lifestyle index” was developed, in which study participants were scored according to lifestyle behaviours and adherence to recognised public health and cancer prevention recommendations [6,20-24] or markers of recommended behaviours. It was hypothesized that a higher score on the healthy lifestyle index would be associated with lower risk of breast cancer.

## Methods

### Study population

The New Zealand Breast Cancer Study, a population-based case–control study, was conducted to investigate risk factors for breast cancer among different ethnic groups in New Zealand. A detailed description of the study design and methods has previously been published [25], and they will therefore only be described briefly here. The study was conducted in three arms comprising Māori, Pacific and non-Māori/non-Pacific women. All women with a primary invasive breast cancer registered on the New Zealand Cancer Registry (NZCR) between 1st April 2005 and 30th April 2006 were eligible for inclusion. To ensure sufficient numbers of cases, the eligible time period was extended for a further year to 30th April 2007 for Māori and Pacific women. Control women were recruited from the New Zealand electoral roll, which has mandatory registration in New Zealand. Controls were matched on ethnicity and frequency matched on 5-year age bands. Consent was obtained from all study participants and ethical approval was granted by the Central Regional Ethics Committee (WGT/03/12/126). The response rate among cases was 78% in non-Māori/non-Pacific women and 81% in Māori; for controls the response rate was 57% in non-Māori/non-Pacific women and 38% in Māori.

### Exclusions

Pacific study participants were excluded due to insufficient numbers for the current analysis (cases n = 70; controls n = 194). Thus, we present here results for Māori and non-Māori/non-Pacific (hereafter referred to as non-Māori) women only. We further excluded cases who completed questionnaires more than one year after the date of their diagnosis of breast cancer (n = 492), since many of the questions in the questionnaire asked participants about their behaviours one year previously. Participants with incomplete diet, lifestyle, and covariate information were also excluded (n = 375). After all exclusions, there were a total of 3211 participants (1093 cases and 2118 controls) included in analyses.

### Data collection and lifestyle factor assessment

All participants completed comprehensive questionnaires on health related behaviours including socio-demographic factors, diet, lifestyle, and reproductive and medical histories. Questions on exercise assessed the average frequency of leisure activities over the preceding year (Godin Leisure Time Exercise Questionnaire) [26,27]. Dietary information was based on questions covering usual number of servings of fruit and vegetables each week; and frequency of red meat, white meat, fish, cream or milk desserts, and cheese consumption over the preceding year. Information on smoking was based on questions regarding current smoking and ever having smoked. Alcohol information included frequency and amount during the preceding year, and at age 20 and 40 years. Body mass index (BMI) was calculated from participants’ self-reported information (weight in kilograms divided by height in metres squared).

Women were classified as premenopausal if they had had a menstrual period in the last three months, or if their periods had stopped due to pregnancy/lactation, or use of hormonal birth control. Women were classified as postmenopausal if they reported not having a period in the last three months, and that this was due to natural menopause, surgical menopause involving bilaterial oophorectomy, or use of hormone replacement therapy (HRT). Women who did not fall into these categories, who reported surgical menopause without bilaterial oophoretoomy, and other or unknown reasons for menses cessation were classified in an ‘other amenorrhea’ category; we then assumed that those aged less than 49 years were premenopausal (n = 85) and those aged 49 years or more were postmenopausal (n = 350), based on data from New Zealand and the UK, which indicate 49 years as the median age at menopause for similar birth cohorts [28,29].

### Lifestyle index score

A healthy lifestyle index score was calculated for each participant based on public health and cancer prevention recommendations [6,20-22,24] (Table 1). Participants reported in an average week last year: how many times a week (none, 1 to 2, 3 to 4, or 5 or more) they ate red meat, white meat, fish, cream, and cheese; how many servings of vegetables (excluding potatoes) they usually ate; how many servings of fruit they usually ate; how many drinks containing alcohol they drank; how many times they did strenuous exercise for more than 15 minutes and moderate exercise for more than 15 minutes; duration of breastfeeding in months for each child; and how many cigarettes they smoked in a day (none, under 10, 10 to 19, or 20 or more). Smoking was categorised as never smoker, ex-smoker, and current smoker. Alcohol consumption was categorised as non-drinker, consumes 1–4 drinks on days that they drink, and 5 or more drinks per occasion across the lifecourse (calculated from responses about consumption during the previous year, consumption at age 40, and consumption at age 20). BMI was categorised into three groups: less than 25 kg/m^2^, 25–29.9 kg/m^2^ (overweight), and 30 kg/m^2^ or higher (obese). Participants scored one point for each reported healthy behaviour derived from considering usual weekly patterns of consumption against recommendations [6,21]. These included: limiting red meat consumption to no more than twice per week; including white meat or fish at least three times; at least 5 portions of fruit or vegetables per day; consuming no cream or cheese; consuming no alcohol; never having smoked; including regular exercise (≥ 36 on the Godin Leisure Score) [27], having BMI of less than 25 kg/m^2^; and cumulative breastfeeding for 6 months or more. Participants received 0.5 points in the intermediate categories of each health behaviour and 0 points for least healthy behaviours. For the analyses, the index score was categorised into tertiles.

**Table 1:** Individual components of the healthy lifestyle index score and their distribution among Māori and non-Māori participants

**Lifestyle factor and index score**		**Māori**			**Non-Māori**			
		**(n = 776)**			**(n = 2435)**			
		**Case**	**Control**		**Case**	**Control**		
		**%**	**%**	**P**	**%**	**%**	**P**	P*
*Recommendation:* Limit intake of red meat								
*Marker:* Red meat								
0	≥5 times per week	20.6	20.8		15.7	16.4		
0.5	3-4 times per week	56.4	48.9		54.2	54.0		
1	≤2 times per week	23.0	30.3	0.213	30.1	29.6	0.894	0.029
*Recommendation:* Choose a variety of protein foods								
*Marker:* White meat								
0	None	–	1.7		3.0	4.2		
0.5	1-2 times per week	58.7	49.7		60.3	61.9		
1	≥3 times per week	41.3	48.6	0.081	36.7	33.9	0.145	<0.001
*Recommendation:* Increase the amount and variety of seafood								
*Marker:* Fish								
0	None	9.5	14.2		12.6	15.3		
0.5	1-2 times per week	74.6	72.8		82.4	78.6		
1	≥3 times per week	15.9	13.1	0.309	5.0	6.1	0.070	<0.001
*Recommendation:* Limit consumption of energy-dense foods								
*Marker:* Cream								
0	≥3 times per week	33.3	32.8		42.3	39.4		
0.5	1-2 times per week	55.6	54.1		43.8	42.5		
1	None	11.1	13.1	0.832	13.9	18.1	0.019	<0.001
*Recommendation:* Reduce the intake of calories from solid fats								
*Marker:* Cheese								
0	≥3 times per week	45.2	44.0		55.4	57.4		
0.5	1-2 times per week	50.8	49.1		41.4	37.0		
1	None	4.0	6.9	0.465	3.2	5.7	0.004	<0.001
*Recommendation:* Eat mostly foods of plant origin								
*Marker:* Vegetables & fruit								
0	<28 servings per week	77.2	76.6		65.6	64.3		
0.5	≥28-< 35	11.9	10.3		13.8	15.8		
1	≥35	11.9	13.1	0.831	20.7	19.9	0.376	<0.001
*Recommendation:* Limit alcoholic drinks								
*Marker:* Alcohol								
0	5+ drinks per time	42.9	39.1		10.3	11.0		
0.5	1-4	42.9	50.3		78.2	79.7		
1	Non-drinker	14.3	10.6	0.242	11.5	9.3	0.221	<0.001
*Recommendation:* Do not smoke								
*Marker:* Smoking								
0	Current	31.0	27.1		8.2	11.7		
0.5	Former	48.4	44.5		36.5	33.8		
1	Never	20.6	28.5	0.191	55.3	54.6	0.017	<0.001
*Recommendation:* Be physically active as part of your everyday life								
*Marker:* Exercise (strenous + moderate Godin score)								
0	<24	61.1	59.1		56.1	54.4		
0.5	≥24-< 36	26.2	19.2		25.5	26.4		
1	≥36	12.7	21.7	0.033	18.4	19.2	0.713	0.002
*Recommendation:* Be as lean as possible without becoming underweight								
*Marker:* BMI								
0	30+	39.7	37.9		21.8	20.7		
0.5	25-< 30	28.6	28.2		33.9	28.5		
1	<25	31.8	34.0	0.878	44.3	50.8	0.004	<0.001
*Recommendation:* Breastfeed infants for at least six months								
*Marker:* Breastfeeding								
0	Never breastfeed	21.4	23.4		25.4	22.2		
0.5	Cumulative breastfeeding >0-< 6 months	21.4	13.1		19.9	17.4		
1	Cumulative breastfeeding ≥6 months	57.1	63.5	0.050	54.7	60.4	0.020	0.046

P value: chi squared test between cases and controls.

P* value: chi squared test between Māori and non-Māori controls.

Recommendation sources: New Zealand Ministry of Health [20-22]; World Cancer Research Fund/American Institute for Cancer Research [6]; U.S. Department of Agriculture and U.S. Department of Health and Human Services [24].

### Covariates

Covariates included were age, parity, age at menarche, history of maternal breast cancer, oral contraceptive use, HRT use, diabetes, and socioeconomic position (SEP). The New Zealand Deprivation Index 2006 [30] was used as a measure of SEP. The Deprivation Index uses nine variables (benefit income, employment, household income, communication, transport, support, qualifications, living space, and home ownership) from the census to place small area blocks on a deprivation scale from 1 to 10; 10 represents the most deprived 10% of New Zealand areas, while 1 represents the 10% least deprived areas. For the analyses, deprivation was categorised into three groups: deciles 1–4 (least deprived), deciles 5–7, and deciles 8–10 (most deprived).

### Statistical analysis

Descriptive analyses were initially conducted to explore the variable values and summarise the data. To compare exposure distributions between cases and controls, chi-squared tests were used for categorical variables and Kruskal-Wallis for continuous variables. Logistic regression was used to estimate the association between breast cancer and the lifestyle index by each menopausal and ethnic group. The lifestyle index was assessed as a categorical variable, adjusted for age at menarche and age at diagnosis/interview as continuous variables, and all other covariates as categorical variables.

Because of the low response rates in the control group, and evidence of differential non-response by deprivation quintile [25], we performed a sensitivity analysis to investigate the possibility of non-response bias by SEP in the controls, using post-stratification weights. A weight was calculated for each stratum of ethnicity*deprivation, by dividing the expected deprivation distribution of each ethnic group by the observed deprivation distribution in the controls from our study. The expected distributions were estimated from the 2002/03 New Zealand Health Survey (unpublished data), and were: 2%, 3%, 10%, 20% and 65% for Māori women in quintiles 1 to 5 of the NZDep2006 categories, and 23%, 20%, 20%, 20% and 17% for non-Māori women. Logistic regression models were then weighted using the “svy: logistic” command in Stata.

All statistical analyses were performed using Stata version 11.2.

## Results

Participants excluded due to missing information were compared to those with complete information; for both Māori and non-Māori, those with incomplete data were less likely to be in the most affluent category and to have ever taken oral contraception. There were 776 Māori women (126 cases, 650 controls) and 2435 non-Māori women (967 cases, 1468 controls) included in the analyses. For cases, the mean time from diagnosis to interview was 247 days. The proportions of cases and controls by all components of the lifestyle index score for each ethnic group are shown in Table 1. Māori women were more likely to eat meat and fish, and less likely to eat cheese than non-Māori women. Fewer Māori also ate the recommended levels of fruit and vegetables, and a higher proportion did not participate in recommended levels of regular exercise. Māori were more likely to drink five or more alcoholic drinks at one time, to be current smokers, and to be classified as obese. Among both ethnic groups, controls were more likely than cases to have breastfed for at least 6 months. Among Māori women the mean healthy lifestyle index score was 5.00; the mean for Māori cases was 4.81 (range: 2.5 to 7.5), and for controls was 5.04 (range: 1 to 9). Among non-Māori women the mean healthy lifestyle index score was 5.43; the mean for cases was 5.39 (range: 1.5 to 9), and for controls was 5.45 (range: 1.5 to 10.5).

There were 987 women classified as premenopausal in the study, including 337 Māori and 650 non-Māori women. The remaining 2224 participants were classified as postmenopausal, including 439 Māori and 1785 non-Māori women. Table 2 shows the distribution of reproductive and lifestyle characteristics by cases and controls, stratified by menopausal status and ethnicity. Among premenopausal Māori women, a history of diabetes was much more frequent in cases than controls, and nulliparity was less frequent. For premenopausal non-Māori women, maternal breast cancer was much more frequent in cases than controls; cases were also more likely to live in deprived areas and experience menarche at a younger age. Statistically significant differences were found between Māori and non-Māori controls for deprivation, parity, age at menarche, and age.

**Table 2:** Distribution of health behaviours and breast cancer risk factors by menopausal status and ethnic group

**Premenopausal**	**Māori (n = 337)**					**Non-Māori (n = 650)**					
	**Case**		**Control**			**Case**		**Control**			
	**(n = 41)**		**(n = 296)**			**(n = 279)**		**(n = 371)**			
	**n**	**%**	**n**	**%**	**P**	**n**	**%**	**n**	**%**	**P**	P*
*Maternal breast cancer*											
No	37	90.2	283	95.6		247	88.5	344	92.7		
Yes	4	9.8	13	4.4	0.141	32	11.5	27	7.3	0.066	0.119
*Oral contraceptive use*											
No	5	12.2	29	9.8		27	9.7	35	9.4		
Yes	36	87.8	267	90.2	0.633	252	90.3	336	90.6	0.917	0.874
*History of diabetes*											
No	33	80.5	279	94.3		271	97.1	359	96.8		
Yes	8	19.5	17	5.7	0.002	8	2.9	12	3.2	0.788	0.114
*Deprivation index*											
Deciles 1–4 (least deprived)	10	24.4	91	30.8		122	43.7	199	53.6		
Deciles 5-7	10	24.4	94	31.8		90	32.3	108	29.1		
Deciles 8–10 (most deprived)	21	51.2	111	37.5	0.241	67	24.0	64	17.3	0.026	<0.001
*Parity*											
Nulliparous	3	7.30	48	16.2		46	16.5	51	13.8		
1-2	23	56.1	117	39.5		152	54.5	192	51.8		
3+	15	36.6	131	44.3	0.093	81	29.0	128	34.5	0.286	0.007
Age at menarche (mean year)	12.8 ± 1.7		12.6 ± 1.6		0.583	12.7 ± 1.5		13.0 ± 1.5		0.017	<0.001
Age (mean year)	43.3 ± 7.1		42.4 ± 6.4		0.272	44.6 ± 5.4		44.9 ± 5.7		0.575	<0.001
**Postmenopausal**	**Māori (n = 439)**					**Non-Māori (n = 1785)**					
	**Case**		**Control**			**Case**		**Control**			
	**(n = 85)**		**(n = 354)**			**(n = 688)**		**(n = 1097)**			
	**n**	**%**	**n**	**%**	**P**	**n**	**%**	**n**	**%**	**P**	P*
*Maternal breast cancer*											
No	77	90.6td	336	94.9		611	88.8	1029	93.8		
Yes	8	9.4	18	5.1	0.129	77	11.2	68	6.2	<0.001	0.440
*HRT use*											
No	63	74.1	274	77.4		445	64.7	730	66.6		
Yes	22	25.9	80	22.6	0.520	243	35.3	367	33.4	0.419	<0.001
*Oral contraceptive use*											
No	29	34.1	91	25.7		219	31.8	242	22.1		
Yes	56	65.9	263	74.3	0.118	469	68.2	855	77.9	<0.001	0.156
*History of diabetes*											
No	69	81.2	302	85.3		625	90.8	1017	92.7		
Yes	16	18.8	52	14.7	0.344	63	9.2	80	7.3	0.158	<0.001
*Deprivation index*											
Deciles 1–4 (least deprived)	9	10.6	95	26.8		254	36.9	560	51.1		
Deciles 5-7	16	18.8	95	26.8		230	33.4	335	30.5		
Deciles 8–10 (most deprived)	60	70.6	164	46.3	<0.001	204	29.7	202	18.4	<0.001	<0.001
*Parity*											
Nulliparous	6	7.1	25	7.1		80	11.6	95	8.7		
1-2	25	29.4	113	31.9		257	37.4	438	39.9		
3+	54	63.5	216	61.0	0.901	351	51.0	564	51.4	0.104	0.007
Age at menarche (mean year)	12.4 ± 1.6		12.7 ± 1.7		0.504	12.9 ± 1.5		12.8 ± 1.4		0.654	0.036
Age (mean year)	59.5 ± 8.3		58.6 ± 7.6		0.432	64.6 ± 10.0		63.5 ± 9.1		0.013	<0.001

P value: chi squared test for categorical variables and kruskal wallace test for continuous variables.

P* value: chi squared test between Māori and non-Māori controls.

Among postmenopausal women, Māori cases were more likely to live in deprived areas than controls. Non-Māori cases were less likely to have used oral contraception, and live in affluent areas; and more likely to have maternal breast cancer than controls. Statistically significant differences were found between Māori and non-Māori controls for HRT, diabetes, deprivation, parity, age at menarche, and age.

The associations between the healthy lifestyle index and breast cancer, adjusted for all covariates, are presented in Table 3 by ethnicity and menopausal status. Compared to the bottom tertile of the healthy lifestyle index score, the top tertile was associated with 31% lower odds of breast cancer in premenopausal Māori women. However, the association did not reach statistical significance. Among postmenopausal Māori women, the top tertile of the healthy lifestyle index score was associated with 53% lower odds of breast cancer when compared to bottom tertile. While this association did reach conventional statistical significance, confidence intervals were very wide, and this result should be interpreted with caution. There was little evidence of an association between the healthy lifestyle index and breast cancer for non-Māori women.

**Table 3:** Adjusted odds ratios (OR) and 95% confidence intervals (CI) for the association between the healthy lifestyle index score and breast cancer by menopausal status and ethnicity

**Healthy lifestyle index score**	**Māori**				**Non-Māori**				
	**Case %**	**Control %**	**OR**	**95% CI**	**Case %**	**Control %**	**OR**	**95% CI**	**P (Ethnic interaction)**
*Premenopausal*	*n = 41*	*n = 296*			*n = 279*	*n = 371*			
T1 (unhealthy score)	46.3	42.2	1.00	Reference	40.9	39.1	1.00	Reference	
T2	34.2	29.4	1.03	(0.47 to 2.26)	27.6	32.6	0.85	(0.58 to 1.25)	
T3 (healthy score)	19.5	28.4	0.69	(0.27 to 1.74)	31.5	28.3	1.23	(0.83 to 1.83)	0.389
P (trend)			0.487				0.371		
Per score unit increase			0.91	(0.69 to 1.20)			1.07	(0.94 to 1.23)	
*Postmenopausal*	*n = 85*	*n = 354*			*n = 688*	*n = 1097*			
T1 (unhealthy score)	50.6	41.8	1.00	Reference	49.7	45.1	1.00	Reference	
T2	34.1	29.4	0.99	(0.56 to 1.73)	28.5	29.1	0.97	(0.77 to 1.22)	
T3 (healthy score)	15.3	28.8	0.47	(0.23 to 0.94)	21.8	25.8	0.86	(0.67 to 1.11)	0.212
P (trend)			0.054				0.262		
Per score unit increase			0.86	(0.70 to 1.05)			0.97	(0.90 to 1.05)	
P (Menopausal interaction)			0.683				0.173		

Adjusted for covariates: parity, age at menarche, history of maternal breast cancer, oral contraceptive use, HRT use, diabetes, SEP, and age.

Weighting controls for differential non-response by deprivation level did not materially alter our results. Compared to the bottom tertile of the healthy lifestyle index score among premenopausal Māori women, the OR for the top tertile decreased from 0.69 to 0.60 (95% CI 0.22 to 1.65). Among postmenopausal Māori women, the OR for the top scoring tertile remained unchanged 0.47 (95% CI 0.23 to 0.95).

## Discussion

This study has found little evidence of an association of the healthy lifestyle index score with breast cancer in non-Māori women, but a moderate association in Māori women, with stronger evidence in those who were postmenopausal than in those who were premenopausal. These results were not explained by accounting for differential non-response in the controls by deprivation levels.

While there are an increasing number of studies on combined lifestyle factors and disease outcomes [14-18], few studies have specifically investigated combined healthy lifestyle indices and breast cancer risk [19,31]. Furthermore, to our knowledge, there are no other studies which have compared the risks associated with a healthy lifestyle index and breast cancer between ethnic groups experiencing different exposure patterns and rates of breast cancer incidence.

The results presented here are novel; however, other factors should also be considered with their interpretation. The study had several limitations, including the potential selection and recall biases commonly associated with case–control studies involving patient interviews. We accounted for non-response in the control group using post-stratification weighting. Exclusion of cases who completed questionnaires after one-year post diagnosis was done to minimise recall bias and reverse causation. Many questions related to behaviours in the previous year i.e. pre-diagnosis and it is likely that those interviewed after one year would have changed behaviour patterns since their cancer diagnosis. Therefore to ensure that the exposures measured were not a direct result of the cancer diagnosis (reverse causation), these participants were excluded from this study.

The study involved relatively small participant numbers. This restricted the levels of categorisation for some variables, and hence, the heterogeneity of the index as there was not enough data for finer stratification. Furthermore, small numbers in some strata, especially among premenopausal Māori women, limit the precision of the effect estimates. However, in light of the different risk profiles for breast cancer by menopausal status these groups were kept separate, rather than producing menopause-adjusted results.

A further limitation was the level of detail available for some exposures, particularly for dietary information. Moreover, the index includes foods that could be inter-related. For example, reducing one’s intake of cheese could be compensated by either increasing consumption of fish (healthy) or red meat (less healthy). The former would receive 2 points associated with healthy behaviours whereas the later would receive only 1 point on the index. The index could also be imprecise for any vegetarians in the study as there was no information available on alternative protein sources. However, the focus of the study was patterns of behaviours rather than specific individual exposures, and the intention was to capture the combined effect of multiple dietary choices and health behaviours. The index components were given equal weight as they were all considered to be indicators of healthy living rather than specific risk factors for breast cancer. The behaviours, or behaviour patterns, are an indication of general lifestyle choices, and measures are likely to reflect habitual exposures, rather than just at a single point in time [32,33]. As carcinogenesis is a lengthy process, it may be preferable to measure all exposures early in life, although it is currently still unknown when the optimal time for this would be, and it is likely to differ by exposure. Therefore the relevance of the exposure measurement at one given time point may depend on the degree to which that exposure tracks over time. There is variable evidence regarding tracking of individual diet and lifestyle factors. In one study, accounting for within person variability in smoking, physical activity and BMI over 20 years of follow up was found to have only a small effect on all-cause mortality risk estimates [34].

While all of the components of the healthy lifestyle index score involve recommended health behaviours, for the dietary components, there is ‘limited’ or ‘suggestive’ evidence with regard to the specific risk associated with breast cancer. The World Cancer Research Fund/American Institute for Cancer Research states this to mean that the current evidence is too limited to permit a probable or convincing causal judgement, but that the evidence shows a generally consistent direction of effect [6].

Based on the healthy lifestyle index score, the healthy behaviours most likely to be followed by participants were breastfeeding, and eating white meat; while the lowest scores were generated for fruit and vegetables, followed by exercise, and cheese consumption. The relative contribution of the components of the index score also differed by ethnic group. Fish consumption generated fewer points for non-Māori, while fruit and vegetables, smoking, and BMI generated fewer for Māori. Overall, our findings suggest that healthy lifestyle interventions could have potential for reducing ethnic inequalities in breast cancer.

The method of cancer detection was not known for the cases in this study (screening or symptomatic); however, the tumour characteristics for the full cohort have been previously published [25], and these show that Māori women had a higher frequency of hormone positive breast cancer than non-Māori. This was also shown in a previous study of more than 21,000 breast cancer cases from the New Zealand Cancer Registry [35]. Consequently, it could be possible that there are differential effects of the HLIS on different subtypes of breast cancer.

Two previous studies have found protective effects for breast cancer associated with a healthy lifestyle, based on different index components. A Mexican case–control study found women in the highest quintile of the calculated healthy lifestyle index had significantly lower odds of developing breast cancer than their counterparts in the lowest quintile of the index (premenopausal OR 0.50, 95% CI 0.29 to 0.84; postmenopausal OR 0.20, 95% CI 0.11 to 0.37) [19]. Their index considered healthy behaviour as being in the lowest tertile of the Western dietary pattern, never consuming alcohol, smoking less than 100 cigarettes, and practicing moderate and vigorous intensity exercise [19]. More recently, a study based on the European Prospective Investigation into Cancer and Nutrition cohort found a lower breast cancer risk for women in the highest category of healthy lifestyle recommendation adherence compared to the lowest scoring category (HR 0.84, 95% CI 0.78 to 0.90) [31]. The components of this index were: body fatness, physical activity, foods that promote weight gain, plant foods, red and processed meat, alcohol intake, and breastfeeding [31]. However the inclusion of body fatness in an index used for combined pre and postmenopausal breast cancer may have attenuated the effect estimate due to the opposite associations between body fat/BMI and pre and postmenopausal breast cancer.

Examination of combined modifiable factors can be an effective way of translating cancer epidemiological findings into primary prevention programmes [17,18,31]. However, further research is needed to properly elucidate healthy lifestyle patterns and cancer risk among different population groups. If the need for lifestyle modification is indeed greatest among specific groups, then ethnic-specific public health interventions may be more efficient to address inequalities. While there is a large body of evidence around public health interventions, there is a paucity of information on differences in uptake among ethnic and socioeconomic groups, and the effects of these on existing disparities. Reviews of tobacco interventions and the effects on social inequalities in smoking have found disadvantaged groups to be more price-sensitive, and hence support tobacco price increases to address inequalities [36,37]. Economic incentives have also been found to have a positive effect on dietary modification and other health related outcomes [38,39]. Māori, Pacific, and low-income New Zealanders often regard healthy food as expensive and unaffordable [40-42], thus fiscal incentives, such as price discounts on fruit and vegetables [43] are likely to be an effective way of addressing health behaviour inequalities.

Ethnic differences have been found in the understanding and interpretation of currently used food labels in New Zealand [40,44]. Disadvantaged groups report not using nutritional labels to help them shop due to lack of time, understanding, and the absence of simple labels on low-cost items [40]. Furthermore, among those using labels, many were still unable to determine whether a food was healthy [44]. Communicating health messages in ways that people can understand is critical to improving behaviour. Information, such as nutritional content, needs to be simplified so that it can be assessed at a glance for consumers.

## Conclusions

These findings suggest that, at a population level, the promotion of healthy lifestyle interventions could have a positive impact on postmenopausal breast cancer risk in Māori women. This study, however, was limited by low numbers, and therefore further work in this area is needed to confirm whether targeted public health intervention programmes would be effective for addressing inequalities in breast cancer incidence.

## Competing interests

The authors declare that they have no competing interests.

## Authors’ contributions

F McK participated in the design of the study, performed the statistical analysis, and drafted the manuscript. LEL participated in the study conception, design, and acquisition of data. MJ contributed to the interpretation of data and helped to draft the manuscript. RF participated in the study conception, design, and acquisition of data. NP contributed to the interpretation of data and helped to draft the manuscript. IR conceived of the study, and participated in its design and helped to draft the manuscript. All authors read and approved the final manuscript.

## Pre-publication history

The pre-publication history for this paper can be accessed here:

http://www.biomedcentral.com/1471-2407/14/12/prepub
